# Supplementary material for: Ready for School: A Multi-Dimensional Approach to School Readiness Assessment in Hispanic Children from Puerto Rico
Source: Behav Sci (Basel). 2025 Jul 15;15(7):957. doi: 10.3390/bs15070957 (PMC12292767; doi:10.3390/bs15070957)
Supplement: Supplementary file 1 [file behavsci-15-00957-s001.zip › behavsci-3677566-supplementary.pdf]

Table S1

Operationalization of Tools/Measures per Domain and Skill Element for School Readiness Assessment of 54 to 65-Month-Old Children

Domain	Elements	Tool	Purpose	Age Range	Scoring Criteria	Scoring	Total Points
Early learning skills (EL)	Letters	W-M: Letter-word identification	Academic strengths and weaknesses across reading, writing, and math assessed by trained examiner. Reliability for the test was estimated at 0.96 – 0.97 (Wendling et al., 2019).	2 – 90 years	Age difference ≤ 12 months	Yes = At Risk = 0<br>No = On track = 1	6 points
	Writing	W-M: Spelling			Age difference ≤ 12 months	Yes = At Risk = 0<br>No = On track = 1	
	Counting	W-M: Applied problems			Age difference ≤ 12 months	Yes = At Risk = 0<br>No = On track = 1	
	Verbal expression	CIF: Do strangers understand child early language?	Child's communication skills reported by parent in clinical assessment.	4.5 – 5.5 years	Positive<br>Negative	Yes = On track = 1<br>No = At Risk = 0	
		CIF: Does your child have difficulties in organizing and expressions ideas?			Positive<br>Negative	Yes = At Risk = 0<br>No = On track = 1	
		CIF: Can he/she re-tell a story in a logical order?			Positive<br>Negative	Yes = On track = 1<br>No = At Risk = 0	
Approach to learning (AL)	Cognitive stimulation	HOME-SF: How often do you read stories to child?<br>- Responses: < 3 times a week = 0<br>- Responses: ≥ 3 times a week = 1<br>HOME-SF: About how many children's books does your child have?<br>- Responses: < 10 books = 0<br>- Responses: ≥ 10 books = 1<br>HOME-SF: Does child have access to a smartphone, tablet, videogames, or computer that allows him/her to listen to music, watch videos or movies, and play games?<br>- Responses: No = 0<br>- Responses: Yes = 1<br>HOME-SF: Do you or have you helped [child] learn numbers/alphabet/colors/shapes and size?<br>- Responses: None = 0	Cognitive stimulation and emotional support in the child's home environment, reported by parent. Instrument reliability range from 0.66 – 0.90 (Bradley and Caldwell, 1979; Mott, 2004).	0 – 15 years	Sum of all cognitive stimulation questions	Total points in 2nd/3rd tertile = On track = 1<br><br>Total points in 1st tertile = At risk = 0	2 points

Domain	Elements	Tool	Purpose	Age Range	Scoring Criteria	Scoring	Total Points
		- Responses: At least 1 = 1<br>HOME-SF: How often does a family member get a chance to take child on any kind of outing?<br>- Responses: Once a month or less often = 0<br>- Responses: 2 – 3 times a month or more often = 1<br>HOME-SF: How often has a family member taken or arranged to take child to any type of museum within the past year?<br>- Responses: Never = 0<br>- Responses: At least once = 1					
	Emotional support	HOME-SF: How much choice is your child allowed in deciding what foods he/she eats at breakfast and lunch?<br>- Responses: Little/no choice = 0<br>- Responses: A great deal/some choice = 1<br>HOME-SF: About how many hours is the TV on in your home each day?<br>- Responses: > 1 hour = 0<br>- Responses: ≤ 1 hour = 1<br>HOME-SF: If child got so angry that he/she hit you, what would you do?<br>- Responses: Any answer implying corporal punishment (i.e., hit/spank the child) = 0<br>- Responses: Non-harm strategies (e.g., talk to the child, send him/her to the room, short time-out) = 1<br>HOME-SF: How often does child eat a meal with you and his/her father/stepfather/father-figure?<br>- Responses: Less often than once a day = 0<br>- Responses: Once a day or more often = 1<br>HOME-SF: About how many times, if any, have you had to spank child in the past week?<br>- Responses: ≥ 2 = 0			Sum of all emotional support questions	Total points in $2^{nd}/3^{rd}$ tertile = On track = 1<br><br>Total points in 1st tertile = At risk = 0	

Domain	Elements	Tool	Purpose	Age Range	Scoring Criteria	Scoring	Total Points
		- Responses: 0 – 1 = 1					
Cognitive skills (CS)	Attention and executive functions	NIHTB-Cog: Flanker	Language, memory, perception, and execution of complex behaviors (control and attention) assessed by trained examiner. Intra-class correlation coefficient (ICC) = 0.76-0.96 (Bauer et al., 2013; Gershon et al., 2013; Zelazo et al., 2013).	3 – 6 years	Standard score <86<br>Standard score 86+	At Risk = 0<br>On track = 1	5 points
		NIHTB-Cog: Dimensional			Standard score <86<br>Standard score 86+	At Risk = 0<br>On track = 1	
	Episodic memory	NIHTB-Cog: Memory sequence			Standard score <86<br>Standard score 86+	At Risk = 0<br>On track = 1	
	Language	NIHTB-Cog: Vocabulary (images)			Standard score <86<br>Standard score 86+	At Risk = 0<br>On track = 1	
	Language development	ASQ-3 Communication	Communication skills, including understanding and expressions (α = 0.66) (Squires et al., 2009).	1 month – 5.6 years	Possible delay in development OR Monitoring zone	At Risk = 0	
					Typical development	On track = 1	
Socioemotional development (SE)	Socioemotional	ASQ:SE-2	Child's socioemotional responses and risk documented by parent. Instrument reliability is 0.90 (Squires et al., 2002).	1 month – 6 years	Professional assessment recommended OR Monitor	At Risk = 0	6 points
					No or low risk	On track = 1	
	Psychological well-being	NIHTB-EM: Positive peer interaction	Child's psychological well-being, social relations, and negative affect documented by parent. Test's reliability ranges from 0.66 - 0.82 (Salsman et al., 2013).	3 – 12 years	T score 0 – 40<br>T score 40+	At Risk = 0<br>On track = 1	
		NIHTB-EM: Positive affect			T score 0 – 40	At Risk = 0	
	Social relationship				T score 40+	On track = 1	
		NIHTB-EM: Empathic behavior			T score 0 – 40<br>T score 40+	At Risk = 0<br>On track = 1	
	Negative affect	NIHTB-EM: Anger			T score 60+<br>T score 0 – 59	At Risk = 0<br>On track = 1	
		NIHTB-EM: Anxiety			T score 60+<br>T score 0 – 5	At Risk = 0<br>On track = 1	
Physical health (PH)	Physical well-being	Clinical assessment by pediatrician	Health and physical problems identified through clinical assessment of medical history and perform comprehensive	4.6 – 5.6 years	Healthy<br>Not Healthy	On track = 1<br>At Risk = 0	6 points

Domain	Elements	Tool	Purpose	Age Range	Scoring Criteria	Scoring	Total Points
			physical and neurological examination by pediatrician.				
	Fine/gross motor skills	ASQ-3: Fine motor	Child's development in communication, gross and fine motor, problem solving, and personal-social skills documented by parent. Test's reliability ranges from 0.72 – 0.83 (Squires et al., 2009).	1 month – 5.6 years	Possible delay in development OR Monitoring zone	At Risk = 0	
					Typical development	On track = 1	
		ASQ-3: Gross motor			Possible delay in development OR Monitoring zone	At Risk = 0	
					Typical development	On track = 1	
		PDMS-2	Performance of age-specific fine and gross motor skills assessed by trained examiner. Instrument reliability range from 0.96 – 0.97 (Folio and Fewell, 2000).	0 – 5 years	Total motor quotient <90	At Risk = 0	
					Total motor quotient 90+	On track = 1	
	Vision screening	Instrument-based vision screener	Amblyopia and risk of vision acuity risks identified with the Welch Allyn Spot Screener (Baxter, n.d.; Donahue et al., 2016). Instrument sensitivity for detecting amblyopia = 89.5%, specificity = 76.7% (Peterseim et al., 2020).	6 months or older	Pass	On track = 1	
					Refer	At Risk = 0	
	Hearing assessment	Audiologist assessment / maternal report	Hearing loss risk assessed by licensed audiologist. Assessment incorporates otoscopy, DPOAEs and ABR based on guidelines of the American Academy of	4.6 – 5.6 years	Pass	On track = 1	
					Fail	At Risk = 0	

Domain	Elements	Tool	Purpose	Age Range	Scoring Criteria	Scoring	Total Points
			Audiology (American Academy of Audiology, 2011).				

Note: W-M = *Batería IV Woodcock-Muñoz: Pruebas de aprovechamiento* (Brief Academic Battery); CIF = Child Investigation Form; HOME-SF = Home Observation Measurement of the Environment – Short Form; NIHTB-Cog = NIH Toolbox Early Childhood Cognition Battery; ASQ-3 = Ages and Stages Questionnaire-3; ASQ:SE-2 = Ages and Stages Questionnaire: Social-Emotional, Second Edition; NIHTB-EM = NIH Toolbox Parent Proxy Emotion Battery; PDMS-2 = Peabody Developmental Motor Scale, Second Edition.

Table S2

Audiological Screening Components: Description, Pass Criteria, and Equipment Used

Procedure	Purpose	Pass Criteria	Equipment / Frequencies	Additional Notes
Otoscopy	Examine the external auditory canal and tympanic membrane	Less than 50% ear canal occlusion by cerumen	Standard otoscope	Medical referral recommended if infection or blockage is suspected
DPOAEs	Assess cochlear (outer hair cell) function	Must meet amplitude and signal-to-noise criteria at each frequency	Senteiro Advance (Path Medical); 2000-5000 Hz	Objective indicator of inner ear function
Pure tone or warble tone audiometry	Assess hearing at specific frequencies	Detect tones at 1000, 2000, and 4000 Hz at 20 dB HL in each ear	Maico MA 25 with headphones; conditioned play audiometry	Child engaged through play-based response strategy
Automated ABR	Assess peripheral vestibulocochlear cranial nerve (CN VIII) integrity	Reliable auditory electrophysiological response to 20 dB eHL chirp stimulus	easyScreen (Maico); alternating polarity stimulus	Child must be calm or asleep for accurate results
Tympanometry	Assess middle ear pressure and mobility	Not performed (equipment unavailable)	N/A	If otoscopy and DPOAEs suggest middle ear effusion, child was referred and re-assessed post-treatment

Note. DPOAEs = distortion product otoacoustic emissions; ABR = auditory brainstem response; HL = hearing level; eHL = estimated hearing level.

Table S3

Clinical Health Criteria Used by Pediatricians to Determine Children's Health Status

Clinical Criterion	Description	Interpretation / Significance	Additional Notes
Number of hospitalizations	Two or more hospital admissions due to chronic or recurrent conditions (e.g., asthma, frequent and severe infections)	Indicates increased risk of repeated hospital admissions due to persistent health issues	Based on medical history
Significant hospitalizations	Serious or prolonged hospitalizations with potential long-term impact (e.g., meningitis, major surgeries)	Suggest possible developmental, sensory, or chronic consequences	Includes repeated or serious surgical procedures
BMI $\geq$ 95th percentile	Defined as obesity, based on CDC age- and sex-specific BMI growth charts	Clinical risk due to elevated weight status	Interpreted using CDC growth standards
Physical findings at 54-65 months	Pediatrician evaluation of chronic or debilitating physical conditions that could interfere with the child's well-being and function	Identification or two or more findings that correlate with chronic or debilitating medical condition<br>Neuromotor findings are significant when correlated with delays on PDMS-2 or ASQ-3 (fine/gross motor domains)	Discrepancies resolved through team pediatricians' consensus, or eternal academic pediatrician review

Note: BMI = body mass index; PDMS-2 = Peabody Developmental Motor Scale, Second Edition ASQ-3 = Ages and Stages Questionnaire-3.

Table S4

School Readiness Assessment Scores by Domain and School Readiness Index (SRI) Composite Scores

Domains	Minimum	Maximum	*M*	*SD*	Q1	Median	Q3
EL	0	6	3.74	1.61	3	4	5
AL	0	2	1.35	0.76	1	2	2
CS	0	5	3.82	1.35	3	4	5
SE	1	6	4.31	1.49	3	5	6
PH	1	6	4.76	1.29	4	5	6
SRI	5	25	17.98	4.46	16	19	21

Note. EL = early learning skills; AL = approach to learning; CS = cognitive skills; SE = socioemotional development; PH = physical health; SRI = school readiness index.

Table S5

School Readiness Results by Domain and Skill Element in Children Ages 54 – 65 Months

Domain / Skill Element	Interpretation	Total	
		n	%
Early learning skills (EL)			
Letters	At-risk	81	68.1
	On-track	38	31.9
Writing	At-risk	43	36.1
	On-track	76	63.9
Counting	At-risk	59	49.6
	On-track	60	50.4
Verbal expression 1 (others understand the child)	At-risk	19	16.0
	On-track	99	83.2
Verbal expression 2 (can organize/express ideas)	At-risk	42	35.3
	On-track	77	64.7
Verbal expression 3 (can re-tell a story logically)	At-risk	22	18.5
	On-track	97	81.5
Approach to learning (AL)			
Emotional support	At-risk	44	37.0
	On-track	75	63.0
Cognitive stimulation	At-risk	34	28.6
	On-track	85	71.4
Cognitive Skills (CS)			
Attention and executive functions (NIHTB-Cog: Flanker)	At risk	19	16.0
	On-track	100	84.0
Attention and executive functions (NIHTB-Cog: Dimensional)	At risk	32	26.9
	On-track	87	73.1
Episodic memory	At risk	55	46.2
	On-track	64	53.8
Language	At risk	8	6.7
	On-track	111	93.3
Language development (ASQ-3 Communication)	At risk	25	21.0
	No or low risk	93	78.2
Socioemotional development (SE)			
Socioemotional (parental perspective)	At-risk	35	29.4
	On track	84	70.6
Psychological well-being	At-risk	25	21.0
	No or low risk	94	79.0
Social relationship (NIHTB-EM: Positive affect)	At-risk	37	31.1
	No or low risk	82	68.9
Social relationship (NIHTB-EM: Empathic behavior)	At-risk	24	20.2
	No or low risk	95	79.8
Negative affect (NIHTB-EM: Anger)	At-risk	34	28.6
	On track	85	71.4
Negative affect (NIHTB-EM: Anxiety)	At-risk	45	37.8

Domain / Skill Element	**Interpretation**	**Total**	
		n	**%**
	On track	74	62.2
Physical health (PH)			
Physical well-being	Unhealthy	37	31.1
	Healthy	81	68.1
Fine/gross motor skills (ASQ-3 Fine)	At-risk	43	36.1
	On track	76	63.9
Fine/gross motor skills (ASQ-3 Gross)	At-risk	23	19.3
	On track	96	80.7
Fine/gross motor skills (PDMS-2)	At-risk	31	26.1
	On track	88	73.9
Vision acuity risk	Refer	20	16.8
	Pass	99	83.2
Hearing loss risk	Refer	0	0.0
	Pass	103	86.6

Note. NIHTB-Cog = NIH Toolbox Early Childhood Cognition Battery; ASQ-3 = Ages and Stages Questionnaire-3; NIHTB-EM = NIH Toolbox Parent Proxy Emotion Battery; PDMS-2 = Peabody Developmental Motor Scale, Second Edition.

Table S6

Correlations Among School Readiness Assessment Domain Scores and School Readiness Index (SRI) Composite Score

Variable	1	2	3	4	5
1. Early learning	-				
2. Approach to learning	.26**	-			
3. Cognitive stimulation	.60**	.24**	-		
4. Socioemotional	.15±	.26**	.20*	-	
5. Physical health	.51**	.17±	.53**	.26*	-
6. School readiness index (SRI)	.77**	.46**	.77**	.57**	.76**

*$p < .05$; **$p < .01$; ± $p < .10$
